# Selection and Engineering of Novel Brighter Bioluminescent Reporter Gene and Color- Tuning Luciferase for pH-Sensing in Mammalian Cells

**DOI:** 10.3390/bios15010018

**Published:** 2025-01-04

**Authors:** Vanessa R. Bevilaqua, Gabriel F. Pelentir, Moema A. Hausen, Eliana A. R. Duek, Vadim R. Viviani

**Affiliations:** 1 Biomaterials Laboratory, Medical and Health Sciences Faculty, Pontifical University Catholic of São Paulo (PUC-SP), Sorocaba 18060-030, SP, Brazil; mahausen@pucsp.br (M.A.H.); eliduek@pucsp.br (E.A.R.D.); 2 Laboratory of Biochemistry, Molecular Biology and Bioluminescent Systems Technology, Department of Physics, Chemistry and Mathematics, Federal University of Sao Carlos (UFSCAR), Rodovia João Leme dos Santos, km 110, Sorocaba 18052-780, SP, Brazil; gabrielpelentir@estudante.ufscar.br (G.F.P.); viviani@ufscar.br (V.R.V.)

**Keywords:** bioluminescence, biosensors, intracellular events

## Abstract

Firefly luciferases have been extensively used for bioanalytical applications, including their use as bioluminescent reporters, biosensors, and for bioimaging biological and pathological processes. Due to their intrinsic pH- sensitivity, in recent years we have demonstrated that firefly luciferases can also be harnessed as color- tuning sensors of intracellular pH. However, it is known that mammalian cells require temperatures higher than 36 °C, which red-shift the bioluminescence spectra of most firefly luciferases, decreasing their activities and the resolution of ratiometric pH analysis. Therefore, we prospected and engineered novel pH-sensitive firefly luciferases for mammalian cells. We humanized the luciferases of *Amydetes vivianii* (Amy-Luc) and *Cratomorphus distinctus* (Crt-Luc) fireflies, inserted them into the pCDNA3 vector, and compared their bioluminescence and pH-sensing properties with those of *Macrolampis* firefly luciferase (Mac-Luc) inside fibroblasts. The transfected COS-1 with Mac-Luc and Crt-Luc displayed lower bioluminescence activity and considerably red-shifted spectra (611 and 564 nm, respectively) at 37 °C, whereas Amy-Luc displayed the highest bioluminescence activity and spectral stability at 37 °C inside cells, displaying the most blue-shifted spectrum at such temperatures (548 nm) and the best spectral resolution at different pH values, making it possible to ratiometrically estimate the pH from 6.0 to 8.0. These results show that Amy-Luc is a novel brighter reporter gene and suitable pH- indicator for mammalian cells. Furthermore, whereas at pH 8.0 the spectrum was thermally stable, at pH 6.0 Amy-Luc showed higher temperature sensitivity, raising the possibility of using this luciferase as an intracellular temperature sensor. Thus, the improved bioluminescence properties as compared to existing luciferases could offer advantages for in vivo imaging and pH- sensing for the study of mammalian cellular physiology.

## 1. Introduction

Alterations in the intracellular pH (ΔpHi) within cells and their organelles can result in the disruption of cell proliferation, ion transport, cellular homeostasis, and potentially cell death [1,2,3,4,5]. Consequently, precisely monitoring the cellular pH is essential. Several fluorescent probes have been developed during the last thirty years aiming to monitor pH dynamics in cells and their organelles [6,7,8,9,10,11,12,13].

Fluorescent indicators are divided into those based on intensity and ratiometric. The intensity-based sensors, or *turn on/off* sensors, are usually simpler, and are based on the increase or decrease in fluorescence intensity upon pH changes but have drawbacks such as the concentration dependency of the probe and a lack of specificity. On the other hand, ratiometric fluorescence sensors are more specific, minimizing the concentration dependency effect of the probe [11,14,15,16,17].

Although fluorescence sensors are more popular, they have disadvantages, including the need for an external excitation source and problems associated with the self-absorption and autofluorescence of the tissue, as well as blanking and phototoxicity [18]. Many fluorescent probes are small molecules with problems associated with diffusion which hamper the proper estimation of the signal inside a single cellular compartment, whereas fluorescent protein probes such as GFP are stable in terms of accumulating in the cells, hampering their use for the real-time monitoring of pH-changes. Furthermore, in the case of photoresponsive tissues, the need for intense irradiation sources to excite fluorescence can be troublesome. For example, in green plant tissue which is highly pigmented and strongly auto-fluorescent, or in the retina and neural tissues that are photoresponsive, the use of intense irradiation sources and generated fluorescence can interfere with these photobiological processes [19,20].

Therefore, pH indicators lacking such drawbacks are in demand, especially for real-time studies of pH dynamics in cells and organelles. In this context, bioluminescent probes are emerging as interesting alternatives for real- time pH estimation.

Bioluminescent probes for pH indication are ratiometric, and are usually based on the Bioluminescence Resonance Energy Transfer (BRET) principle. They usually involve the fusion proteins of a luciferase or photoprotein with a pH-sensitive GFP variant [21]. Zhang et al. (2012) developed a ratiometric pH probe named “pHlash” that utilizes BRET, thereby avoiding excitation by an external light source [22]. However, the authors point out two main weakness: the lower luminescence intensity generated by bioluminescence, and the stability of the signal catalyzed by the luciferase. Despite avoiding the problems associated with fluorescence sensors, BRET-based bioluminescent sensors may have the disadvantage of using larger fusion proteins whose interaction could be affected by other factors.

Firefly luciferases have been already extensively used for bioanalytical applications, including their use as bioanalytical reagents for ATP assays, as bioluminescent reporters for biosensors, and for imaging biological and pathogenic processes such as tracking metastasis, pathogenic bacteria, and viral infections as well as development of high throughput drug screening assays [23,24]. Beetle luciferases have the additional advantage of eliciting different colors, varying from green to red.

Whereas firefly and other beetle luciferases emitting different bioluminescence colors have been extensively used in dual- and triple- reporter assay systems [25], no use of their intrinsic pH- and metal sensitivity has been put forward until recently. We first showed that a single firefly luciferase can be harnessed as a color- tuning intracellular sensor of pH in bacteria and mammalian cells [26,27], and as an enzymatic sensor for toxic metals [28,29]. The main proton binding site responsible for pH- sensitivity has been identified, involving the electrostatic interactions between E311 and R337, and between H310 and E354, that keep a closed active site conformation favorable for green emission at alkaline pH values, and is broken at acidic pH values opening and polarizing the active site, resulting in red light emission [30,31]. Firefly luciferases also display spectral sensitivity to temperature [32]. Unfortunately, for mammalian cells which work at higher temperatures, firefly luciferases are usually less stable and display already quite red- shifted spectra [33], decreasing the magnitude of the pH-mediated shift and therefore the resolution of the ratiometric analysis of pH.

To overcome these drawbacks, herein we compared the suitability of two novel pH-sensitive firefly luciferases developed in our laboratory which display different bioluminescence colors and pH- sensitivities as reporter genes and intracellular pH-sensors in mammalian cells. For this purpose, we constructed pCMV vectors containing the codon-humanized cDNAs *Cratomorphus distinctus* (pCMV- Crt) and *Amydetes vivianii* (pCMV-Amy) firefly luciferases [34,35], and used the previously constructed *Macrolampis* firefly luciferase (PCMV-Mac) to transfect COS-1 cells and compare their suitability as reporter genes and as bioluminescence color- tuning pH indicators in mammalian cells.

## 2. Materials and Methods

### 2.1. Plasmid Construction and Luciferase Gene Engineering

The luciferase of the *Macrolampis* sp2 firefly was previously subcloned into the pCMV vector [33]. The luciferase genes of the *Amydetes viviani* and *Cratomorphus distinctus* fireflies were codon- humanized (GenOne, Rio de Janeiro, Brazil) and then ligated into the Hind III site of the pCMV plasmid to generate pCMV-Amy and pCMV-Crt. Maxi-preps of these DNAs were prepared using Wizard^®^ Plus Maxiprep DNA Purification System (Promega, Madison, WI, USA).

### 2.2. Cell Culture

An amount of 2.5 × 10^5^ COS-1 cells was previously seeded in a 6-well plate, or 5 × 10^4^ cells in a 24-well plate, depending on the experiment. The cells were kept in DMEM at a high concentration of glucose with L-glutamine and phenol red (DMEM) supplemented with 10% fetal bovine serum (Cultilab, Campinas, SP, Brazil) and 1% penicillin/streptomycin/amphotericin in an incubator, with a humidified 5% CO_2_ atmosphere at 37 °C. COS-1 cells were acquired by Rio de Janeiro Cell Bank (BCRJ, cell type code 070 batch number 000114).

### 2.3. Transfection

The COS-1 cells were transfected with 0.5 to 1.5 µg of the plasmids pCMV-Mac, pCMV-Amy, and pCMV-Crt/well, using the Lipofectamine^TM^ 3000 (Invitrogen- Thermo Fisher, São Paulo, Brazil) reagent following the manufacturer’s instructions.

### 2.4. Comparison of In Vivo Bioluminescence Activity

During the second day after the transfection, we added 10 µL of 100 mM D-luciferin to adhered confluent COS cells and measured their bioluminescent activity (cps) for 120 s, using a multiusuary NightOwl Bioluminescence Photodetection Camera (Berthold Technologies GmbH & Co. KG, Bad Wildbad, Germany). The bioluminescent activity was monitored using the same equipment, at time intervals of 40 min between the images, for a period of 12 h. The graph of activity versus time was plotted as a function of the relative activity of the luciferases.

We also visually compared the bioluminescent activity using images obtained from a photographic camera adapted to a handmade black Styrofoam box. For this experiment, the transfected COS-1 cells were then trypsinized and removed from the plate, centrifuged, and resuspended in a calibration buffer (135 mM Potassium phosphate, 1 mM MgCl_2_, 1 mM CaCl_2_, 20 mM NaCl, 10 mM Glucose, 2 μg/mL Nigericin). Subsequently, D-luciferin was added to the suspension and the effect of pH on the bioluminescent intensity and color was photographed, using a Canon Camera (São Paulo, Brazil, Iso: 12000; exposure time: 30 s).

### 2.5. The Effect of pH on the Bioluminescence Spectra

To measure the effect of pH on the spectra of cells at room temperature (22 °C), COS-1 cells transfected with pCMV-Amy, pCMV-Crt, and pCMV-Mac were removed from a 6- well plate and incubated in calibration buffer consisting of a 135 mM potassium phosphate buffer containing 1 mM MgCl_2_, 1 mM CaCl_2_, 10 mM glucose, and 20 mM NaCl, in the presence of a 2 µg/mL nigericin ionophore. Bioluminescence spectra were measured using an AB-1850 spectroluminometer (ATTO, Tokyo, Japan) provided with a cooled CCD camera. To measure the spectra, 90 µL of the cell solution was mixed with 10 µL of 10 mM D-luciferin. The reported spectra are the average result of three independent experiments. The estimated peak error is ±2.5 nm.

### 2.6. The Effect of Temperature on the Bioluminescence Spectra

To measure the bioluminescence spectra at different temperatures of the purified Amy luciferase and COS-1 cells transfected with pCMV-Amy, we used a Hitachi F4500 spectrofluorometer (Hitachi, Tokyo, Japan) using the following parameters: excitation shutter closed; emission window slit = 20 nm; scanning wavelength = 450–700 nm and scanning speed = 2400 nm/min; photomultiplier voltage = 400 V, coupled with a thermostatic bath (Bioplus IT-2002- incubator time) at 22, 37, and 45 °C, at different pH values (pH 6.0, 6.5, 7.0, 7.5, and 8.0). The reported spectra were the average result of three independent experiments. The estimated peak error is ± 2.5 nm.

In the case of *Amydetes* purified luciferase, the luciferase was expressed in bacteria and purified by Nickel-agarose affinity chromatography according to Pelentir et al. (2020) [29]. In the assay, 170 µL of 0.10 M Sodium Phosphate buffer (pH 6.0, 7.0 or 8.0) were mixed with 10 µL of purified Amy luciferase (0.5 mg/mL), 10 µL of 10 mM of D- luciferin and 10 µL of a mixture of 80 mM MgSO_4_ and 40 mM ATP, totaling 200 µL of sample.

To measure the effect of pH on the spectra of COS- 1 transfected cells at different temperatures, the cells were removed from a 6- well plate previously incubated in calibration buffer consisting of 135 mM potassium phosphate buffer containing 1 mM MgCl_2_, 1 mM CaCl_2_, 10 mM glucose, and 20 mM NaCl, in the presence of 2 µg/mL nigericin ionophore. For in vivo spectra measurements, 180 µL of resuspended cells at different pH levels (pH 6.0, 7.0, and 8.0) were mixed with 20 µL of 10 mM of D- luciferin, also totalizing 200 µL of sample. The number of cells used to measure the spectra varied from 2.5 × 10^5^ to 5 × 10^5^ according to the conditions.

### 2.7. Ratiometric Analysis at Room Temperature

The ratio (R = I_green_/I_red_) for COS-1 cells transfected with pCMV-Amy, pCMV-Crt, and pCMV-Mac was determined using an AB-1850 spectroluminometer (ATTO, Japan) at room temperature (22 °C). The effect of pH was plotted as a function of the R values. The emission peak of the spectra for the green emission at pH 8.0 and red at pH 6.0 were determined for each luciferase and used to obtain the ratio of bioluminescence intensities in the green and red regions (I_Green_/_Red_):(pCMV-Amy = I_551_/I_583_; pCMV-Crt = I_564_/I_583_, pCMV-Mac = I_583_/I_612_).

### 2.8. The Effect of Temperature on the Ratiometric Analysis

We also analyzed the effect of temperature on the green/red light intensities for COS-1 cells transfected with pCMV-Amy. For this purpose, we used a F4500 spectrofluorometer (Hitachi, Japan) coupled with a thermostatic bath. The cells (200 µL) were pre-incubated, as previously described in the topic 2.6, in the spectrofluorometer cell for 5 min, until the desired temperature was reached, before the spectral scanning. Using this equipment, the non-corrected bioluminescence emission peaks for the green and red light emissions for Amy-Luc transfected cells were 542 and 608 nm (R = I_542_/I_608_).

### 2.9. Photographic Detection

Photographic images of bioluminescent cells were taken with a Ti5 Cannon camera (parameters: Iso 12000, exposure time of 30 s, smaller shutter opening).

## 3. Results and Discussion

### 3.1. Comparison of BL of Fibroblasts Expressing Different Firefly Luciferases

Previously, COS-1 cells transfected with pCMV plasmid harboring *Macrolampis* sp2 firefly luciferase (pCMV-Mac) showed for the first time that a firefly luciferase could be used as an intracellular pH indicator in mammalian cells [33]. However, the spectrum of this luciferase (λ_max_ = 611 nm) as well as the spectra of most firefly luciferases are considerably red- shifted at 37 °C (λ_max_ > 600 nm), decreasing the magnitude of the pH-mediated shift and, therefore, the potential resolution of ratiometric analysis. Therefore, herein we prospected the use of two new green light emitting luciferase cDNAs from *Cratomorphus distinctus* (λ_max_ = 564 nm) and *Amydetes vivianii* fireflies (λ_max_ = 550 nm), which display different colors and pH- sensitivities [34,35], in COS-1 cells. These luciferases cDNAs were codon- humanized and inserted into the pCMV vector to generate pCMV-Crt and pCMV-Amy.

**Figure 1:**
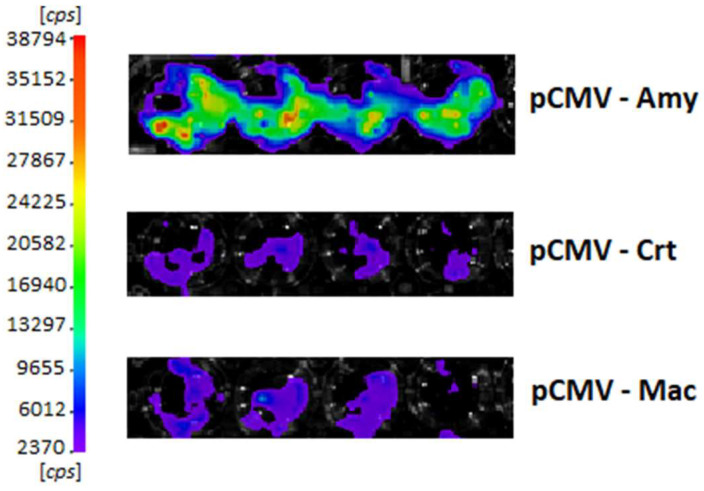
Comparison of the bioluminescent activity of four sets of COS-1 cells transfected with pCMV vector harboring the pH-sensitive luciferase genes, after the addition of D-luciferin, showing the highest intensity for cells transfected with pCMV-Amy. Photography taken with Cannon Camera (Iso: 12000; exposure time: 30 s).

**Figure 2:**
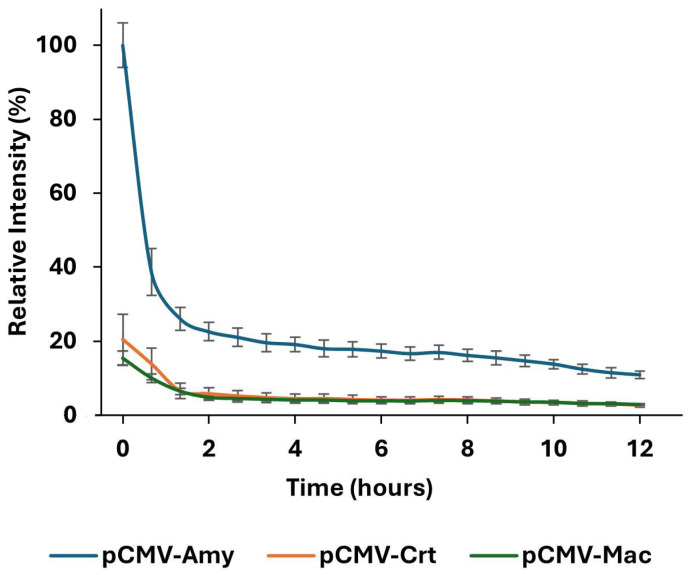
The bioluminescent kinetic profile of COS-1 cells transfected with pH-sensitive luciferase genes for 12 h after the addition of D-luciferin, showing a higher relative intensity for cells transfected with pCMV-Amy. The error bars refer to triplicates performed for the experiment.

The cells transfected with pCMV-Crt displayed low bioluminescence activity and bioluminescence spectrum in the yellow- green region (λ_max_ = 564 nm), whereas cells transfected with pCMV-Amy displayed the highest activity inside COS-1 cells (Figure 1) and the most blue-shifted spectrum (λ_max_ = 548 nm). The bioluminescence intensity half-life was ~30 min, and they decayed to ~10% of the initial intensity after 12 h (Figure 2). Cells transfected with pCMV-Amy showed at least five-fold higher activity than those transfected with pCMV-Crt or pCMV-Mac (Figure 2).

### 3.2. The Comparison of the Effect of Intracellular pH Changes with Different Luciferases

**Figure 3:**
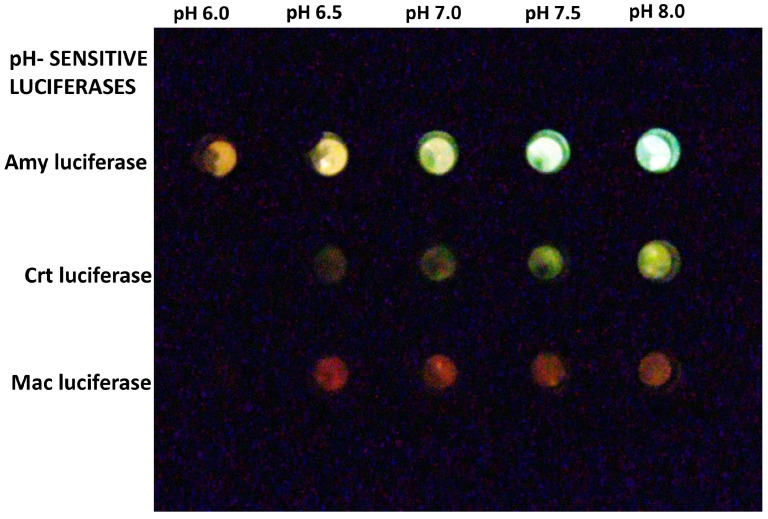
Bioluminescent COS-1 cells transfected with pCMV containing different pH-sensitive firefly luciferases at different pHs after incubation in calibration buffer containing nigericin. Cells transfected with pCMV-Amy luciferase showed the most intense bioluminescence and slight color change at different pH.

COS-1 cells transfected with different luciferases were incubated in calibration buffer containing the proton transporter nigericin in order to equilibrate the extracellular and intracellular pH. The cells transfected with pCMV-Amy displayed a higher bioluminescent intensity and a larger color difference at different pHs, when compared to cells transfected with Crt and Mac luciferases (Figure 3). Cells transfected with pCMV-Mac exhibited reddish bioluminescence at all pHs. The higher bioluminescent intensity of cells transfected with Amy luciferase allowed photographic detection with a conventional Canon camera, or even with a cell phone camera in a dark environment.

**Figure 4:**
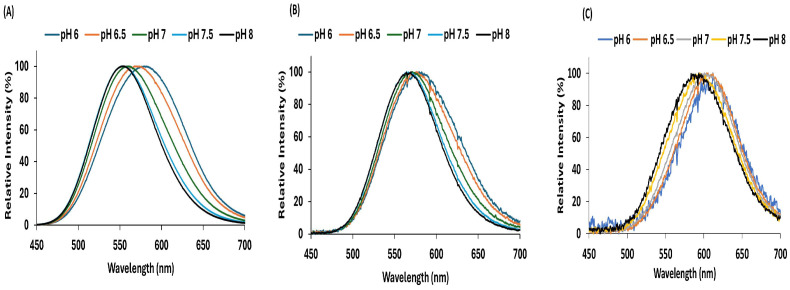
The effect of pH on the bioluminescence spectra of COS-1 cells transfected with plasmid pCMV containing firefly luciferases: (**A**) pCMV-Amy; (**B**) pCMV-Crt; (**C**) pCMV-Mac.

We then compared the bioluminescence spectra, the emission peaks and bandwidths of these different luciferases at different pHs. The spectral shift (ΔλpH_6–8_) and the bandwidth [half-band] caused by these pH changes, were higher for *Amydetes* luciferase (26 and 24 nm, respectively), and lower for Crt (10 and 19 nm, respectively) and Mac (16 and 6 nm, respectively) (Figure 4 and Table 1).

### 3.3. Ratiometric Analysis

The green/red light intensity ratio at different pH values from 6.0 to 8.0 was analyzed using the spectroluminometer. Then, the linear regression lines of the ratios at different pHs were obtained for the cells transfected with the three different luciferases and the angular coefficients were calculated (Table A1 and Table 2). The angular coefficient is the inclination of the curve, and the higher the coefficient, the higher the inclination and, therefore, the larger the color change per unit of pH.

**Figure 5:**
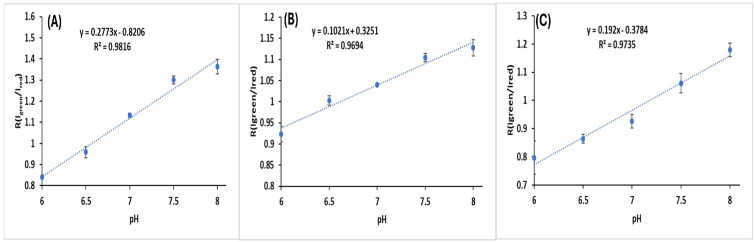
Ratiometric curves showing the effect of pH on the ratio of green and red light intensities (R = I_green_/I_red_) obtained from the spectra measured in the spectroluminometer: (**A**) pCMV-Amy (R = I_551_/I_583)_; (**B**) pCMV-Crt (R = I_564_/I_583_); (**C**) pCMV-Mac (R = I_583_/I_612_). The standard deviations are shown in the Table A1.

Cells transfected with Amy-luciferase showed a higher angular coefficient (0.28) and greater spectral variation from pH 6.0 to 8.0 (Δλ = 26 nm), as well as a larger half-band broadening, followed by the Mac (0.19) and Crt (0.10) luciferases (Figure 5).

### 3.4. Effect of Temperature and pH on BL Spectra of Fibroblasts Transfected with AmyLuc

We next conducted a detailed investigation of the bioluminescent properties of the pH-sensitive Amy luciferase expressed in COS-1 cells (pCMV-Amy) and compared these properties to those observed for the purified enzyme (pCold-Amy). This comparison accounts for the potentially distinct microenvironments experienced by the luciferase inside the cell versus in vitro assay conditions. We compared the bioluminescence activity and spectra of these cells and of the purified luciferase at different temperatures and at different pHs.

**Figure 6:**
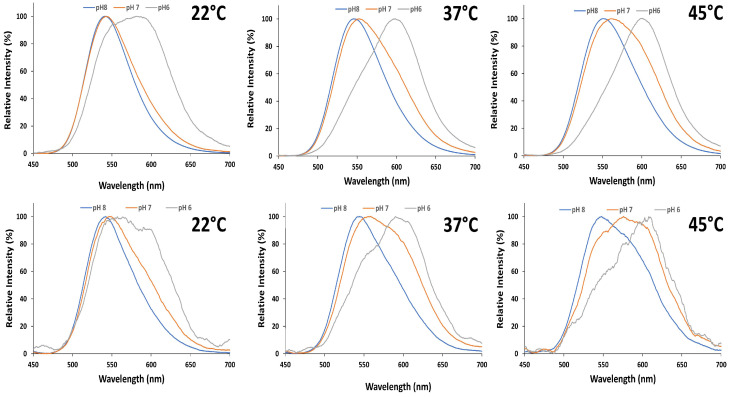
The effect of pH on the bioluminescence spectra at different temperatures: (**upper** horizontal panel) purified *Amydetes vivianii* luciferase; (**lower** panel) COS-1 cells transfected with pCMV-Amy.

At 22 °C, upon varying the pH from 8.0 to 6.0, the purified luciferase and the pCMV- Amy transfected cells displayed a red-shift (Δλ) of 40 and 15 nm in the emission peak, respectively, with a broadening of the bandwidth indicating an increase in the red- light emission (Figure 6 and Table 3 and Table 4). At 37 °C, upon the same pH variation, the purified enzyme and transfected cells displayed a red- shift of 52 and 46 nm, respectively. At 45 °C this displacement increased to 48 and 60 nm, respectively.

**Figure 7:**
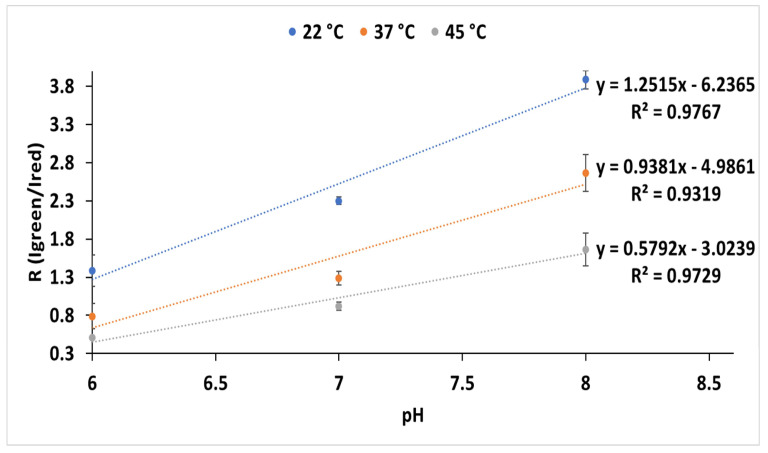
Ratiometric analysis of the effect of pH on green (I_550_) and red (I_600_) emissions (RI_550_/I_600_) at different temperatures: 22 °C (blue); 37 °C (orange); 45 °C (gray). The standard deviation ranged from 2 to 10%.

The ratio between green (λ_max_ = 542 nm) and red (λ_max_ = 608 nm) light intensities in COS-1 cells transfected with this luciferase (pCMV-Amy), showed a larger increase in red- light emission at higher temperatures (Figure 7). The larger spectral shifts at 37 and 45 °C in relation to 22 °C indicate that, although this luciferase bioluminescence spectrum is more stable and less sensitive to pH at lower temperatures under in vitro conditions, it is a much better pH indicator at the elevated temperatures of mammalian cells.

### 3.5. Potential Use of Amy Luciferase as Temperature Sensor

Previously, we showed that firefly luciferases display different temperature sensitivities that could be potentially useful for temperature sensing [32]. However, no applicability of such sensitivity had yet been prospected. Considering that, herein we also analyzed the possibility of using Amy-Luc as a temperature sensor. Whereas this luciferase is less pH-sensitive and temperature- sensitive of the studied firefly luciferases, at higher temperatures it manifests a higher spectral sensitivity. At pH 6.0, the increase in the temperature from 22 to 45 °C caused a red- shift of 17 nm for the purified enzyme, and 51 nm for the transfected COS-1 cells. At pH 7.0, these values were 18 and 28 nm, respectively. Finally, at pH 8.0, the spectral displacements were only 9 and 6 nm, respectively (Table 4). These results indicate that Amy-Luc is thermally more stable at alkaline pH values, which is in agreement with recent results that show that the secondary structure scaffold of this enzyme is thermally more stable at pH 8.0 than that at pH 6.0 [36]. It is possible that the larger spectral change observed at 37 and 45 °C for the cells, in relation to the luciferase in vitro, could be caused by additional acidification and stress inside the cells at such high temperatures. Therefore, although this luciferase is not such a good sensor for temperature at alkaline pH values, due to its intrinsic thermal stability, its higher thermal sensitivity at pH < 7.0 reveals a potential applicability as a temperature sensor at lower pHs.

### 3.6. Smartphone Detection

**Figure 8:**
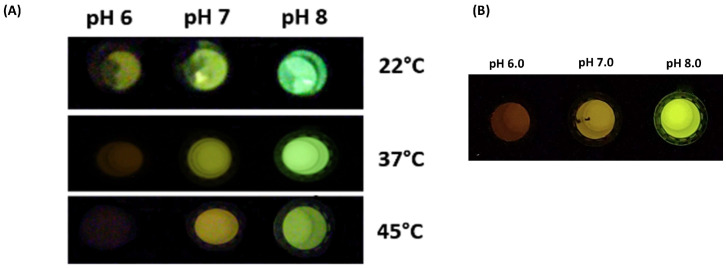
The photographic detection of bioluminescent COS-1 cells transfected with pCMV-Amy: (**A**) the effect of pH on the bioluminescence color of COS-1 cells transfected with pCMV-Amy at different temperatures. Photography taken with Cannon Camera (Iso: 12000; exposure time: 30 s; (**B**) the effect of pH on the bioluminescence color at room temperature. Photography taken with an Edge 20 pro smartphone (Motorola).

The bioluminescence intensities of COS-1 cells transfected with pCMV-Amy at different pH values (Figure 8A) and at different temperatures ranging from 22 to 45 °C (Figure 8B) were intense enough to allow the photographic detection of different colors with a smartphone camera. The possibility of photographic detection with smartphone cameras also opens the possibility of future applications of this system for *hands- on* biosensors in *point- of- care* applications, such as we have recently shown for the bioluminescence color detection of cadmium (personal communication).

## 4. Concluding Remarks

The comparison of bioluminescence of fibroblasts transfected with pCMV constructs harboring three different pH-sensitive firefly luciferases showed that *Amydetes* luciferase (AmyLuc) displayed the best bioluminescence properties as a reporter gene and as pH-ratiometric indicator for mammalian cell bioimaging at 37 °C. AmyLuc displayed a bioluminescent intensity at least five- fold higher than the other used firefly luciferases, as well as higher stability at 37 °C, allowing bioluminescence detection not only with spectrometers, but also with smartphone CCD cameras. The more blue-shifted and less sensitive spectrum of this luciferase to pH at lower temperatures becomes more sensitive at higher temperatures, providing a pH indicator better suited to mammalian cells. Furthermore, the higher temperature spectral sensitivity at acidic pH values of this enzyme also reveals a new potential applicability of this luciferase to temperature sensing inside organelles such as mitochondria.

## Figures and Tables

**Table 1:** The effect of pH on the emission peaks and half-band width of the bioluminescence spectra of COS-1 cells transfected with pCMV plasmid containing firefly luciferases genes and the resultant spectral shifts measured between pH 6.0 and 8.

Luciferase	λ_max_ [Half-Band] (nm) *					
	pH 6.0	pH 6.5	pH 7.0	pH 7.5	pH 8.0	Δλ_(pH6–8)_ (nm)
pCMV-Amy	579 [111]	570 [109]	561 [99]	553 [91]	553 [87]	26 [24]
pCMV-Crt	579 [103]	576 [99]	571 [90]	564 [86]	569 [84]	10 [19]
pCMV-Mac	604 [93]	605 [93]	597 [96]	588 [99]	588 [99]	16 [6]

* The average peak error is ±2.5 nm.

**Table 2:** Comparison of the angular coefficients of the ratiometric curves for the different luciferases.

Angular Coefficient
Amy > Mac > Crt
0.28 > 0.19 > 0.10

**Table 3:** Bioluminescence spectral properties of purified *Amydetes vivianii* luciferase and in COS-1 cells transfected with pCMV-Amy.

Luciferase	λ_max_ [Half-Band] (nm)								
	22 °C			37 °C			45 °C		
	pH 6.0	pH 7.0	pH 8.0	pH 6.0	pH 7.0	pH 8.0	pH 6.0	pH 7.0	pH 8.0
pCold-Amy (purified)	581 [107]	543 [74]	541 [67]	598 [91]	552 [93]	546 [75]	598 [85]	561 [102]	550 [82]
pCMV-Amy (COS-1)	557 [108]	548 [86]	542 [70]	590 [101]	558 [106]	544 [82]	608 [101]	576 [105]	548 [98]

**Table 4:** Temperature-dependent variation in the bioluminescence spectra emission peaks (Δλ) of purified *Amydetes vivianii* luciferase and transfected COS-1 cells at different pH values.

Luciferase	Δλ (nm)					
	ΔT_22–45°_ (°C)			ΔpH _6.0–8.0_		
	pH 6.0	pH 7.0	pH 8.0	22 °C	37 °C	45 °C
pCold—Amy (purified)	17	18	9	40	52	48
pCMV—Amy (COS-1)	51	28	6	15	46	60

## Data Availability

Data will be available upon request.

## Appendix A

**Table A1:** Ratiometric analysis for bioluminescent COS1 cells transfected with different luciferases.

Luciferase		pH 6	pH 6.5	pH 7	pH 7.5	pH 8	Linear Regression Line
pCMV-Amy							Y= 0.2773x − 0.8206
	Average	0.84	0.95	1.13	1.30	1.36	
	Standard deviation	0.008	0.026	0.009	0.018	0.0348	
pCMV-Crt							Y = 0.1021x + 0.3251
	Average	0.92	1.00	1.03	1.10	1.12	
	Standard deviation	0.018	0.011	0.003	0.01	0.018	
pCMV-Mac							Y = 0.192x − 0.3784
	Average	0.79	0.86	0.92	1.06	1.17	
	Standard deviation	0.01	0.014	0.032	0.029	0.03	
